# Intravenous Administration of Heat Shock-Treated MSCs Can Improve Neuroprotection and Neuroregeneration in Canine Spinal Cord Injury Model

**DOI:** 10.3390/ani10112164

**Published:** 2020-11-20

**Authors:** Woo Keyoung Kim, Wan Hee Kim, Oh-Kyeong Kweon, Byung-Jae Kang

**Affiliations:** 1 Department of Veterinary Clinical Sciences, College of Veterinary Medicine and Research Institute for Veterinary Science, Seoul National University, Seoul 08826, Korea; wkkimcoca@snu.ac.kr (W.K.K.); whkim@snu.ac.kr (W.H.K.); ohkweon@snu.ac.kr (O.-K.K.); 2 BK21 FOUR Future Veterinary Medicine Leading Education and Research Center, Seoul National University, Seoul 08826, Korea

**Keywords:** spinal cord injury, mesenchymal stem cells, heat shock response, canine, neuroregeneration

## Abstract

**Simple Summary:**

Mesenchymal stem cells (MSCs), which are found in connective tissues, can be used to treat spinal cord injury (SCI) in dogs. These stem cells have the ability to repair damaged tissues and can be transplanted into the injured area. While this is considered a promising treatment, the transplanted cells often do not survive in the injured spinal cord. In this study, we found that heat shock treatment, i.e., exposure to high temperatures, increased the efficacy of MSC treatment for SCI.

**Abstract:**

Transplantation of mesenchymal stem cells (MSCs) is a promising treatment for spinal cord injury (SCI). However, many transplanted cells die within a few days, eventually limiting the efficacy of cellular therapy. To overcome this problem, we focused on the potential of heat shock (HS) proteins in facilitating recovery from cell damage and protecting against cytotoxicity. PCR results showed that the expression of neurotrophic factor, anti-inflammatory, stemness, and homing genes increased in HS-treated MSCs. We investigated whether HS-treated MSCs could promote recovery of hindlimb function in an acute canine SCI model. We compared the effects of intravenous transplantation with (i) lactated Ringer’s solution as a control, (ii) green fluorescent protein-expressing MSCs (MSCs-GFP), and (iii) GFP-expressing and HS-treated MSCs (MSCs-GFP-HS). Spinal cords were harvested at four weeks and used for Western blot and histopathological analyses. The MSCs-GFP-HS group showed significant improvements in hindlimb function from weeks 3 and 4 compared with the other groups. This group also showed higher expression of neural markers, fewer intervening fibrotic changes, and pronounced myelination. These results suggest that induction of an HS response in MSCs could promote neural sparing. In conclusion, transplantation of HS-treated MSCs could improve neuroprotection and neuroregeneration in acute SCI.

## 1. Introduction

Traumatic spinal cord injury (SCI), such as contusion and compression, disrupts neural circuits, resulting in sensory and motor problems [1,2]. Following SCI, regeneration of the central nervous system is hampered by various factors such as tissue damage, disruption of the axon, and inhibitory molecules [3,4]. Interestingly, preserved tissue in injured areas has been suggested to contribute to the repair of damaged neural circuits [2]. This spontaneous recovery is limited, however, because only a small number of host neurons remain in the injured spinal cord [5,6]. From this viewpoint, transplantation of exogenous neurons to injured areas of the spinal cord would be an ideal approach for treating SCI.

As a treatment for SCI, stem cell transplantation is considered a promising method [7]. Previous studies have shown that transplantation of mesenchymal stem cells (MSCs) after SCI can induce neural differentiation and improve functional recovery [8]. However, many transplanted cells die within the first few days because of the hostile environment, which reduces the effectiveness of cell therapy [9,10,11]. There is an urgent need to develop methods to overcome this problem.

Heat shock response (HSR) is a homeostatic reaction that acts on many stress factors. Activation of HSR induces the expression of heat shock proteins (HSP), which facilitate recovery from cell damage [12,13,14]. Several studies have shown that HSR and HSP expression is debilitated in neurons of the central nervous system [15,16]. Decreasing the HSR in differentiated neural cells contributes to vulnerability to the pathological environment caused by stress [17]. Furthermore, heat shock (HS) treatment confers protection against cytotoxicity when differentiated cells are stressed or challenged [18]. Therefore, HS treatment is a promising approach to improve the viability of transplanted cells.

In this study, we hypothesized that HS-treated MSCs improve cell survival, neuroregeneration, and neurological function in an acute canine SCI model. In addition, we used intravenous (IV) administration methods in a non-invasive way to evaluate the migration degree and efficacy of MSCs. The findings of this study might aid in the development of an effective treatment approach for SCI.

## 2. Materials and Methods

### 2.1. Animal Selection

The experiment was conducted in 12 healthy 1- to 2-year-old male beagles, with an average weight of 10.63 ± 0.88 kg. All dogs had clinically good health and were neurologically normal. Dogs were assigned to one of three groups (four dogs per treatment): (i) control, IV administration of 20 mL lactated Ringer’s solution only; (ii) MSCs-GFP, IV administration of 1 × 10^7^ MSCs expressing green fluorescent protein (GFP); and (iii) MSC-GFP-HS, IV administration of 1 × 10^7^ HS-treated MSCs expressing GFP. During the experiment, all dogs were cared for in accordance with the Animal Care and Use Guidelines (The Institute of Laboratory Animal Resources, Seoul National University, Korea). The study was approved by the Animal Care and Use Committee of Seoul National University (SNU-190401-7, SNU-181214-3).

### 2.2. Isolation and Culture of MSCs

In other dogs that are not assigned to this experimental group, gluteal fat tissue was aseptically sampled under general anesthesia. The anesthesia of the dog was carried out as follows: cefazolin sodium (40 mg/kg IV, Cefazoline; Chong Kun Dang Pharm), tramadol (4 mg/kg IV, Toranzin; Samsung Pharm. Ind. Co., Seoul, Korea), acepromazine (10 μg/kg IV, Sedaject; Samu Median Co., Seoul, Korea), and alfaxalone (2 mg/kg IV; Alfaxan; Jurox). Anesthesia was maintained with 2% isoflurane (Ifran; Hana Pharm. Co., Seoul, Korea). Electrocardiography, rectal temperature, pulse oximetry, and respiratory gas analysis were measured using the monitoring system (Datex-Ohmeda S/5; GE Healthcare, Little Chalfont, UK). The collected fat tissues were cleaned with phosphate-buffered saline (PBS) and homogenized. Next, the samples were digested using collagenase type-1 (1 mg/mL; Sigma-Aldrich, St. Louis, MO, USA) for 2 h in a shaking incubator. The digested fatty tissue was centrifuged at 220× *g* for 5 min after being filtered through a 100 mm pore nylon mesh. After removing the supernatant, the stromal vascular fraction (SVF) was carefully separated. The SVF was resuspended and cultured in a 150 mm culture dish with low-glucose Dulbecco’s modified Eagle’s medium (DMEM; GenDEPOT, Grand Island, NY, USA), 10% fetal bovine serum (FBS; Gibco BRL, Grand Island, NY, USA), and 1% penicillin and streptomycin (PS) at 37 °C with 20% O_2_ and 5% humidified CO_2_. Twenty-four hours later, cell debris and unattached cells were washed with PBS. The medium was changed every 48 h until the cells reached 80–90% confluence. In this experiment, the third passage of cells was used as an allograft. These cells had previously been identified as having multilineage differentiation capabilities [19,20].

### 2.3. Green Fluorescent Protein (GFP) Labeling of the MSCs

To track stem cells, MSCs were transfected with GFP-coded lentivirus vectors. We used the Dharmacon Trans-Lentivirus packaging system to make transgenic viruses (GE Healthcare, Lafayette, CO, USA). Briefly, HEK293T cells (Invitrogen, Carlsbad, CA, USA) were cultured in a 100 mm culture dish in high-glucose medium (DMEM) containing 10% FBS and 1% PS. When the cells reached 90% confluence, pCDH-EF1-MCS-pA-PGK-copGFP-T2A-Puro vector (System Biosciences, Mountain View, CA, USA) was mixed with lentiviral packaging mix (Fisher Scientific Cat#14-432-23) encoding the viral proteins Gag-Pol, Rev, and VSV-G, and was transfected into cells for lentivirus production. After the cells were incubated for 48 h with 20% O_2_ and 5% CO_2_ at 37 °C, GFP-expressing virus particles were collected from the culture media. The first passage MSCs were transduced with lentiviruses at MOI 100, when they reached 50–60% confluence. After the MSCs reached 90% confluence, the selection process was carried out using puromycin (3 μg/mL; Gibco-BRL). Finally, we obtained MSCs expressing GFP. The cells were cultured to passage three, which were then used in the experiments.

### 2.4. Heat Shock Treatment

For heat shock treatment, third passage MSCs expressing GFP were cultured with 20% O_2_ and 5% CO_2_ at 37 °C. Once the cells reached 90% confluence, they were moved to a 43 °C incubator and incubated for 1 h. After 1 h, the cells were moved back to the 37 °C incubator and underwent a 3 h recovery process [21].

### 2.5. Real-Time Quantitative PCR (RT-qPCR)

mRNA was isolated from MSCs-GFP and MSCs-GFP-HS using QIAzole (Qiagen, Hilden, Germany) solution according to the manufacturer’s protocol. RNA purity and concentration were determined using Gen 5.2 reader type, Epoch (BioTek, Winooski, Vermont, VT, USA). cDNA was synthesized using the Prime Script II First-Strand cDNA Synthesis Kit (TaKaRa, Otsu, Japan). RT-qPCR was performed using the ABI StepOnePlus Real-Time PCR system (Applied Biosystems, Foster City, CA, USA) by mixing SYBR Premix Ex Taq (TaKaRa) and the specified primers (Table 1). The mRNA expression was normalized with GAPDH and quantification for comparison of gene expression was performed using the 2^−ΔΔCT^ method [22].

### 2.6. Spinal Cord Injury Induction

The previously described balloon compression method was used to induce experimental SCI [23]. The anesthesia was carried out in the same way as mentioned above and hemilaminectomy was performed at the fourth lumbar segment (L4). Through fluoroscopic guidance, an 8 Fr silicone Foley catheter (Yushin, South Korea) was inserted through a hole in L4 and placed at the cranial margin of the first lumbar segment (L1). The balloon was inflated by inserting 50 μL/kg of contrast agent (Omnipaque, Amersham Health, Carrington Hill, Ireland) by diluting it with normal saline (1:1) (Video S1). The Foley catheter was fixed using the Chinese finger trap suture, and six hours after recovering from the anesthesia, it was carefully removed. To relieve pain after surgery, tramadol (4 mg/kg IV, Toranzin; Samsung Pharm. Ind. Co., Seoul, Korea) and local anesthesia with lidocaine (2 mg/kg) and bupivacaine (2 mg/kg) mixture (1:1) were carried out. In a dog that felt discomfort when it woke up after surgery, medetomidine (2 μg/kg IV, Domitor; Zoetis, Korea) was given to the dog additionally. The dogs showed complete paraplegia and had no sensory or motor reflexes. For postoperative care, cefadroxil (22 mg/kg PO twice daily; Ilyang pharm., Korea) was administered for three days and tramadol (4 mg/kg PO 3 times a day, Tridol Cap.; Yuhan, Korea) for seven days. To prevent the pressure sore from sitting in the same position for a long time, we applied soft padded bandage to dogs and monitored them in an intensive care unit. Water intake, feed intake, body temperature, pulse rate, and respiration rate were carefully managed every day. Furthermore, manual bladder pressure was applied for urination at least three times a day.

### 2.7. Intravenous Administration of MSCs

After removal of the catheter, injection of lactated Ringer’s solution, MSCs-GFP, or MSCs-GFP-HS was performed. In the control group, 20 mL of lactated Ringer’s solution was injected intravenously for three consecutive days. In the case of the MSCs-GFP, and MSCs-GFP-HS groups, the third passage cells were harvested at 90% confluence. When the cells reached 90% confluence, washed them twice with PBS and incubated with 0.05% trypsin-EDTA (Sigma-Aldrich, St. Louis, MO, USA) for 15 min at 37 °C and 5% CO_2_. Then, the cells were centrifuged at 220× *g* for 5 min and pelleted. The cell pellet mixed with 1 mL lactated Ringer’s solution and used a Countess FL Automated Cell Counter (Thermo Fisher Scientific, Pittsburg, PA, USA) after staining with Trypan blue to measure the number of cells. Approximately 1 × 10^7^ MSCs-GFP and 1 × 10^7^ MSCs-GFP-HS, respectively, were diluted with 20 mL of lactated Ringer’s solution and administered intravenously for three consecutive days. The dogs were carefully observed for four weeks after the injections.

### 2.8. Sample Preparation

After four weeks of experimentation, all dogs were euthanized. The anesthesia for euthanasia proceeded as follows: tramadol (4 mg/kg IV, Toranzin; Samsung Pharm. Ind. Co., Seoul, Korea), acepromazine (10 μg/kg IV, Sedaject; Samu Median Co., Seoul, Korea), and alfaxalone (2 mg/kg IV; Alfaxan; Jurox). Anesthesia was maintained with 2% isoflurane (Ifran; Hana Pharm. Co., Seoul, Korea). The dogs were euthanized by alfaxalone (2 mg/kg IV; Alfaxan; Jurox) and bolus injection of 10 mL KCl solution (1 M) into the cephalic vein. The injured spinal cord was collected and fixed in 10% sucrose for 12 h at 4 °C and moved to 20% sucrose at 4 °C for approximately 24 h. The dura mater was removed from the spinal cord and embedded in an optimal cutting temperature compound (Rica Biosystems, Richmond, VA, USA) and frozen using liquid nitrogen. Embedded spinal cords were cut longitudinally to form two parts: one was used for western blot analysis and the other was used for immunofluorescent staining and histopathological assessment.

### 2.9. Western Blot Analysis

Only the injured spinal cord was collected, frozen with liquid nitrogen, and smashed into small pieces with a hammer. After 30 min of incubation with RIPA lysis buffer (Gen Depot, Grand Island, NY, USA) and proteinase inhibitor solution (Gen Depot), centrifugation was performed for 15 min at 12,000× *g* at 4 °C and the upper layer was separated. The Bradford assay was used to determine the protein concentration in the supernatant. A protein of 20 μg was separated with 10% sodium dodecyl sulfate-polyacrylamide gel electrophoresis (SDS-PAGE) and transferred to a polyvinylidene difluoride (PVDF) membrane. The membrane was blocked for 1 h with 5% skim milk and incubated overnight with primary antibodies. The following primary antibodies were used: nestin (neural progenitor stem cells, sc-23927), glial fibrillary acidic protein (GFAP, astrocytes, sc-33673), and β-III-tubulin (immature neurons, sc-80005). Afterwards, the membrane was incubated with anti-mouse secondary antibody (sc-516102) for 1 h, and the protein band was visualized using enhanced chemiluminescent substrates (ECL) (Bio-Rad, Hercules, CA, USA) and quantified using an LAS 4000 mini system (GE Healthcare, Lafayette, CO, USA).

### 2.10. Immunofluorescence Assessment

With a thickness of 10 μm, the spinal cord was cut longitudinally using a cryotome, mounted on silane-coated slides, fixed for 10 min with 4% paraformaldehyde, and permeabilized with 0.1% *v/v* Triton X-100 for 3 min. The slides were blocked with 10% FBS for 1 h and then incubated overnight with primary antibodies for nestin (sc-20978), GFAP (sc-65343), and β-III-tubulin (sc-69966) at 4 °C. The slides were then incubated for 2 h with fluorescein iso-thio-cyanate conjugated anti-mouse (Alexa flour, ab-150111) and anti-rabbit (Flamma 648) secondary antibodies. DAPI (4,6-diamidino-2-phenylindole) staining was conducted to stain the nuclei, which were observed using a microscope (EVOS FL Imaging System, Stanwood, WA, USA). Cells that were positive for specific markers were randomly counted in five injured areas, and the value was expressed as a percentage of 1000 cells.

### 2.11. Histopathological Assessment

Hematoxylin and eosin (H&E; Thermo Fisher Scientific, Waltham, MA, USA) and Luxol fast blue (LFB; American MasterTech, Lodi, CA, USA) staining were carried out in accordance with the manufacturer’s instructions to identify demyelination, fibrosis, vacuole formation, and hemorrhage. We performed histomorphometric analyses using a computer-associated image analysis system (Image-J version 1.52a; National Institute of Health, Bethesda, MD, USA). To quantify the staining regions, the ratio of the staining area to be evaluated was presented as an average and statistically compared.

### 2.12. Behavioral Assessment

Behavioral assessments were conducted for four weeks after surgery for hindlimb motor function assessment. During the observation, dogs were allowed to move freely for five minutes in a confined area and hindlimb coordination, weight-bearing ability, and locomotion were recorded. One of the dogs that could not be weighed alone was supported by the experimenter by holding the lower part of the tail. Neurological function was objectively evaluated using the 19-point scoring system for canines of Basso, Beattie, and Bresnahan (cBBB) (Table 2) [24] and revised and modified Tarlov scales (Table 3) [25]. Three experimenters conducted evaluations of the dogs’ gait with blinded experimental conditions, and the score was expressed as an average for each group.

### 2.13. Statistical Analysis

Data were analyzed using SPSS software (version 23 IBM, Chicago, IL, USA), and all data are expressed as mean ± standard deviation. A non-parametric Kruskal–Wallis test, was performed with Mann–Whitney U post-hoc test. A *p* value of 0.05 or less was considered to be statistically significant.

## 3. Results

### 3.1. GFP Expression and Heat Shock Treatment Characteristics of MSCs

**Figure 1:**
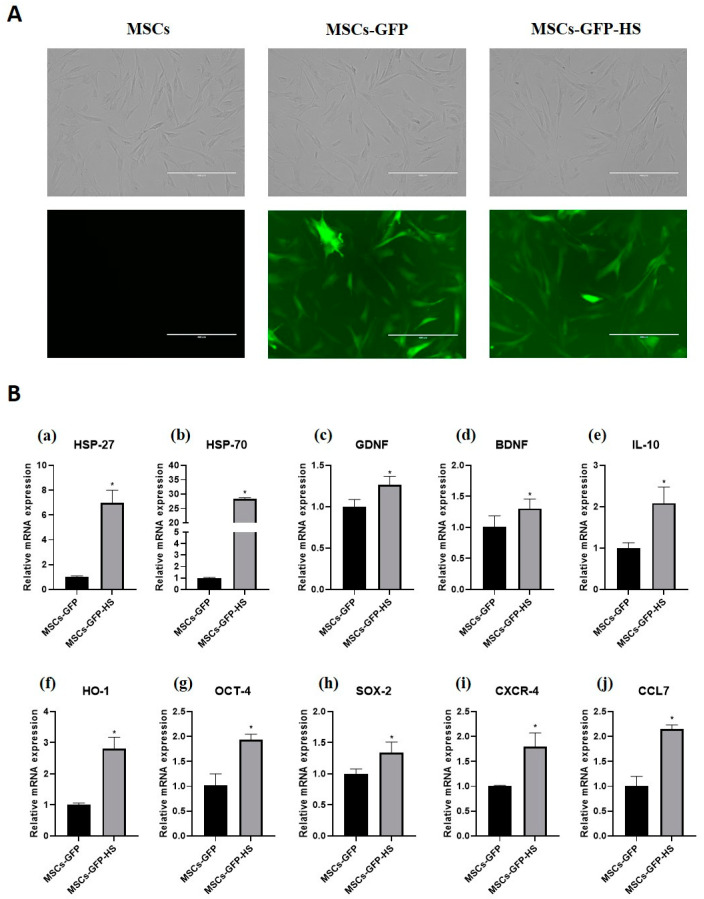
GFP labeling and heat shock-treated mesenchymal stem cells (MSCs). (**A**) Expression of GFP was identified in the MSCs-GFP and MSCs-GFP-heat shock (HS) groups using a fluorescence microscope. All cells showed fibroblast-like morphology. (**B**) mRNA expression of (**a**) HSP-27, (**b**) HSP-70, (**c**) glial cell-derived neurotrophic factor (GDNF), (**d**) brain-derived neurotrophic factor (BDNF), (**e**) IL-10, (**f**) HO-1, (**g**) OCT-4, (**h**) SOX-2, (**i**) CXCR-4, and (**j**) CCL7. The scale bar indicates 400 μm. The data were obtained by experiments repeated three times; each bar represents average of the gene expression calculated with the formula 2^−ΔΔCT^ and was normalized to the MSCs-GFP group. Error bars represent standard deviation. * denotes significance at *p* ≤ 0.05.

When observed through a fluorescence microscope, the MSCs-GFP and MSCs-GFP-HS expressed green fluorescence and all cells showed similar morphology to fibroblasts (Figure 1A). Heat shock treatment was identified by the genes related to heat shock response. Heat shock protein 27 (HSP-27) and heat shock protein 70 (HSP-70) were highly upregulated in the MSCs-GFP-HS group (Figure 1a,b, *p* < 0.05). The expression of neurotropic factors, such as glial cell-derived neurotrophic factor (GDNF) and brain-derived neurotrophic factor (BDNF), was upregulated in the MSCs-GFP-HS group (Figure 1c,d, *p* < 0.05). Regarding the expression of anti-inflammatory gene markers, interleukin 10 (IL-10) and heme oxygenase 1 (HO-1) were significantly upregulated in the MSCs-GFP-HS group compared with that in the MSCs-GFP group (Figure 1e,f, *p* < 0.05). Comparing the expression levels of stemness-related genes, octamer-binding transcription factor 4 (OCT-4) and SRY-box transcription factor 2 (SOX2) were upregulated in the MSCs-GFP-HS group (Figure 1g,h, *p* < 0.05). The homing factors C-X-C chemokine receptor type 4 (CXCR-4) and C-C motif chemokine ligand 7 (CCL7) were significantly upregulated in the MSCs-GFP-HS group (Figure 1i,j, *p* < 0.05).

### 3.2. Histopathological Assessment

**Figure 2:**
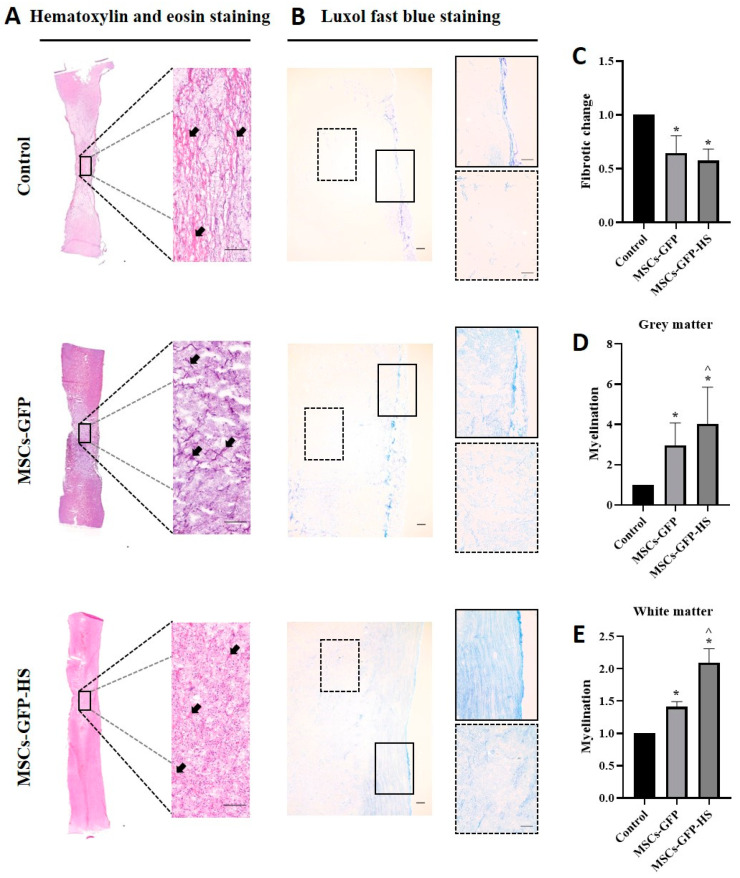
Histopathological analysis of the stained injured spinal cords. (**A**,**B**) Images of the injured spinal cord sections of the control, MSCs-GFP, and MSCs-GFP-HS groups. The sections were stained with hematoxylin and eosin (H&E) and Luxol fast blue (LFB) to identify fibrotic changes, hemorrhages, vacuolar formation, parenchymal change, and degree of myelination. Scale bar represents 200 μm for all images. In the LFB staining image, the lined box is the magnified image of the grey matter and the dotted box is the magnified image of the white matter. (**C**) Quantitative comparison of the fibrotic changes is shown as red and pink (arrow) in the H&E staining. (**D**,**E**) Quantitative comparison of the grey and white matter myelin level, which is depicted by the intensity of the blue color. (**C**–**E**) Each bar indicates the average of four samples per group and was normalized to the control group. Error bars represent standard deviation. * denotes significance compared with the control group at *p* ≤ 0.05. ^^^ denotes significance compared with the MSCs-GFP group at *p* ≤ 0.05.

Histopathological analysis of H&E staining revealed pathological variation in the spinal cord parenchyma. Fibrotic changes, atrophic changes, vacuolar formation, and hemorrhages in the injured spinal cord were identified in all samples. At low magnification of the spinal cord lesions, the MSCs-GFP-HS group showed a well-organized parenchymal matrix, but the other groups showed a more distorted parenchymal matrix (Figure 2A). Fibroblast-like cell proliferation, hemorrhage, and vacuolar formation of the injured area at high magnification were also observed to be reduced in the MSCs-GFP-HS group. Fibrotic areas were less intervened in the MSCs-GFP-HS group and the MSCs-GFP group than in the control group (Figure 2A, arrow). Quantification of fibrotic changes with H&E staining showed there were significant differences between the control group and MSCs-GFP group (Figure 2C, *p* < 0.05) and between the control group and MSCs-GFP-HS group (Figure 2C, *p* < 0.05).

LFB staining was used to identify myelination of the injured spinal cord. Injured lesions contained vacuoles, which exhibited loss of neurons, oligodendrocytes, astrocytes, and myelination. Compared with the MSCs-GFP group and the MSCs-GFP-HS group, the control group displayed severe demyelination. In contrast, the MSCs-GFP-HS group exhibited significantly improved demyelination (Figure 2B). Based on the quantification of myelinated area, the MSCs-GFP-HS group showed pronounced myelination of gray matter and white matter by enhanced LFB staining (Figure 2D,E, *p* < 0.05).

### 3.3. Immunohistochemical Assessments and Western Blot Analysis

**Figure 3:**
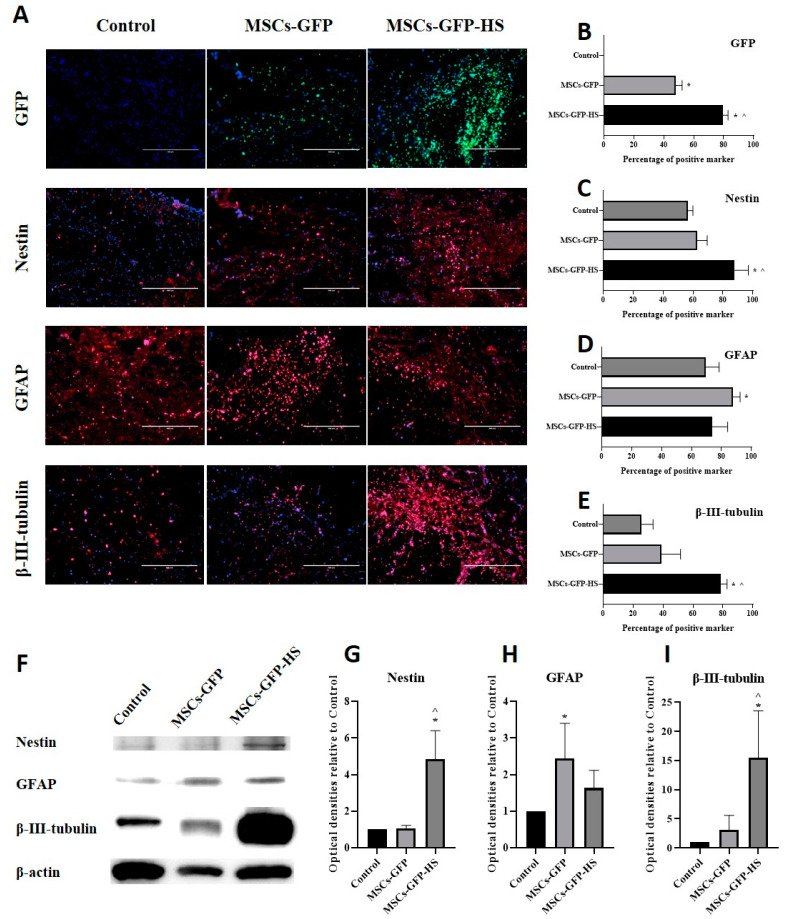
Immunohistochemical assessments and Western blot analysis at four weeks after transplantation. (**A**) Injured spinal cord lesions were stained with nestin, GFAP, and β-III-tubulin as red; transplanted cells (green fluorescent protein (GFP]); the nucleus was stained with DAPI as blue. Scale bar represents 400 µm for all images. (**B**) Percentage of GFP positive cells. (**C**–**E**) Percentage of neural marker positive cells. (**F**) Representative densities of neural markers. (**G**–**I**) Quantitative analysis of densities obtained for nestin, GFAP, and β-III-tubulin normalized to the control. Each bar indicates the average of four samples per group. Error bars represent standard deviation. * denotes significance compared to the control group at *p* ≤ 0.05. ^^^ denotes significance compared to the MSCs-GFP group at *p* ≤ 0.05.

Immunohistochemistry was performed to identify whether cells transplanted into injured spinal cord regions had migrated and to evaluate the expression of neural markers. The presence of GFP expression in the injured spinal cord region confirmed the successful migration of stem cells to the injured spinal cord after IV injection (Figure 3A). The percentage of GFP positive cells was significantly higher in the MSCs-GFP-HS group than in the control (Figure 3B, *p* < 0.05) and MSCs-GFP groups (Figure 3B, *p* < 0.05). Most of the cells expressing GFP were distributed around the lesion of the SCI, especially in the cranial and caudal margins. The percentage of nestin-expressing cells was significantly higher in the MSCs-GFP-HS group than in the control group (Figure 3C, *p* < 0.05) and MSCs-GFP group (Figure 3C, *p* < 0.05). The percentage of β-III-tubulin positive cells was also significantly higher in the MSCs-GFP-HS group than in the control group (Figure 3E, *p* < 0.05) and the MSCs-GFP group (Figure 3E, *p* < 0.05). However, the percentage of GFAP positive cells in the MSCs-GFP group was higher than that in the control group (Figure 3D, *p* < 0.05) and MSCs-GFP-HS group. Compared to the MSCs-GFP group, the percentage of GFAP positive cells in the MSCs-GFP-HS group was higher.

A similar pattern of marker expression in the IHC results was identified in the western blot results (Figure 3F). The expression of nestin and β-III-tubulin was highest in the MSCs-GFP-HS group. Nestin (Figure 3G, *p* < 0.05) and β-III-tubulin (Figure 3I, *p* < 0.05) expression were significantly higher in the MSCs-GFP-HS group than in the other groups. As shown by immunohistochemistry, the expression of GFAP was significantly higher in the MSCs-GFP group (Figure 3H, *p* < 0.05). The expression of GFAP in the MSCs-GFP-HS group was lower than that in the MSCs-GFP group and higher than that in the control group.

### 3.4. Behavioral Observations

**Figure 4:**
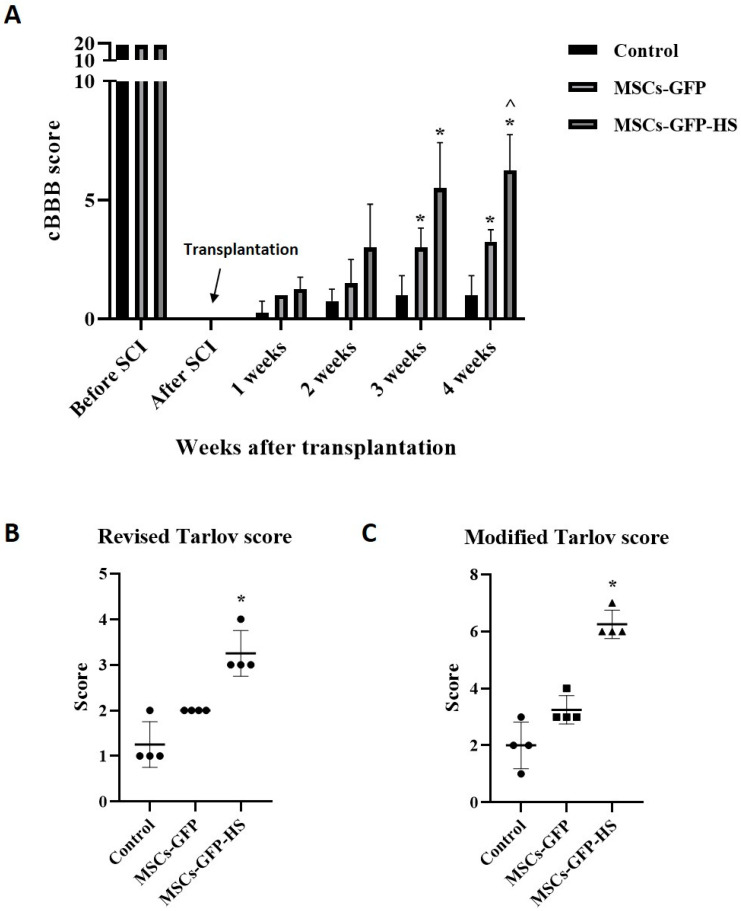
Behavioral analysis using canine Basso, Beattie, and Bresnahan (cBBB) scores and Tarlov scales until four weeks. (**A**) Improvements in cBBB scores before and during the four weeks following transplantation. Hindlimb locomotion at four weeks after transplantation were measured by two grading systems: (**B**) revised Tarlov scales and (**C**) modified Tarlov scales. Error bars represent standard deviation. * denotes significance compared with the control group at *p* ≤ 0.05. ^^^ denotes significance compared with the MSCs-GFP group at *p* ≤ 0.05.

The cBBB score of the experimental dogs was 19 before the SCI and decreased to zero after SCI. The dogs’ hind limbs showed complete paralysis, with no pain. Scores were measured weekly for up to four weeks after transplantation. All groups showed progressive motor function recovery after cell transplantation, especially in the MSCs-GFP-HS group. From the third week onwards, the cBBB scores showed significant differences between groups, with the fourth week being the most obvious. In the third and fourth weeks, the cBBB score was significantly higher in the MSCs-GFP-HS group than in the control group (Figure 4A, *p* < 0.05). Compared with the MSCs-GFP group, the MSCs-GFP-HS group achieved the highest score in week 4 (Figure 4A, *p* < 0.05). In the MSCs-GFP-HS group, all dogs were able to stand; three dogs showed full ambulation and one dog showed limited ambulation. Even though coordination of the legs was poor, the dogs could support their own weight. In the MSCs-GFP group, only one dog was able to stand with assistance and three dogs had a limited ability to stand, but they showed purposeful hindlimb motion. In the control group, only one dog showed purposeful hindlimb motion, and three dogs showed flaccid tone in the hindlimbs. To qualitatively evaluate these behaviors, neurological recovery was evaluated using the revised and modified Tarlov scales four weeks after transplantation (Figure 4B, C, *p* < 0.05).

## 4. Discussion

Many strategies have been used to treat SCI. One strategy is stem cell transplantation into the injured spinal cord. Stem cell transplantation has the ability to repair damaged neuronal tissues and provide therapeutic agents. Among stem cells, adipose-derived mesenchymal stem cells (Ad-MSCs) can be easily acquired, rapidly expanded, and induce neuronal differentiation and neuroregeneration. IV-delivered Ad-MSCs have been reported to promote functional recovery of acute SCI in dogs [26]. However, transplanted cells lack viability because damaged spinal cords do not provide an ideal environment. Even under optimal microenvironmental conditions, only approximately 10% of stem cells survive in the injured central nervous system [27,28,29], and very few cells differentiate into mature neuronal phenotypes [30,31,32]. There are reports of ways to improve the treatment efficiency of stem cell therapy by manipulating the genes of stem cells. However, gene editing has the potential risk of causing genetic mutations [33,34]. In contrast, the HSR is not genetically modified but is evolutionarily preserved and maintains homeostasis under various stresses [35].

HSPs are protective proteins against various stresses [35]. Heat shock transcription factors (HSFs) are activated in response to various stresses, inducing transcription of HSPs [36]. The upregulation of HSP expression through HS treatment has been shown to have a cytoprotective effect that resists stresses such as oxidative stress and hypoxia, inhibits apoptotic pathways, and inhibits proinflammatory cytokines [35,37,38]. In the present study, we used a 43 °C incubation period to produce HSP-expressing MSCs that resist various external stresses. HS-treated MSCs were successfully produced, as evidenced by the increased expression of genes related to the HSR. In addition, growth factors, antioxidants, and stemness factors increased. These results indicate that HS-treated MSCs possibly resisted the hostile microenvironment in injured spinal cords and induced neuroregeneration.

In this study, we injected stem cells during the acute phase of SCI. After SCI, the blood-spinal cord barrier is destroyed, resulting in cell infiltration, inflammatory reactions, and pathological changes in lesions. This change activates astrocytes to create a glial scar, creating an environment that interferes with neural regeneration [8,39,40]. Hence, it is crucial to control inflammation and the microenvironment by administering stem cells early in SCI for neuronal protection. Previous reports showed that MSCs intravenously injected into an acute SCI canine model had antioxidant and anti-inflammatory effects [26]. Our results showed that expression of the anti-inflammatory and antioxidation-related genes was higher in the HS-treated MSCs group than in the MSCs-GFP group. We found that the HS-treated group had the lowest fibrosis of the lesion and more neuronal sparing. Considering the cytoprotective, anti-inflammatory, and antioxidant effects of HS-treated MSCs, these results were likely because of proper control of the inflammatory response that occurs in the early stages of SCI.

Previous studies indicated that stem cells migrate to the lesion of the spinal cord following IV administration [26,41]. In this study, the results also showed that the IV-administrated stem cells were distributed around the injured spinal cord, most of which were in the cranial and caudal margins. The distribution of migrated stem cells did not differ between the MSCs-GFP and MSCs-GFP-HS groups. In studies using mouse and rat models, IV-transplanted cells were not detected in the injured lesion six weeks later [42], and cells transplanted into the subarachnoid space were not observed four weeks later [43]. Therefore, a large number of cells were thought to be needed for IV; hence, the injections were administered for three consecutive days in this study. This repeated administration of stem cells appears to have caused many cells to migrate to the injured spinal cord, which were marginally distributed four weeks later, and improved neuronal sparing in the lesion. In addition, given that there were more cells expressing GFP in the HS-treated MSCs group compared with the other groups, many transplanted cells appear to have migrated to the site of the injury, possibly due to the high expression of homing-related genes (CXCR-4, CCL7) in HS-treated MSCs.

Reactive astrocytes protect the lesion in SCI and contribute to healing of the surrounding area, which has two contrasting aspects because it prevents nerve regeneration of the lesion [44]. Therefore, it is important that the reactive astrocytes remain at appropriate levels for protection and regeneration. In a prior study, the transplantation of MSCs to the central nervous system showed activation of astrocytes [45]. In accordance with this result, GFAP expression in the MSCs-GFP group was higher than that of the control group and the MSCs-GFP-HS group, but myelination in the MSCs-GFP group was relatively lower than that in the MSCs-GFP-HS group. It appears that transplanted MSCs-GFP enhanced the inhibitory properties of reactive astrocytes. On the other hand, the MSCs-GFP-HS group showed lower GFAP expression than the MSCs-GFP group but higher than that of the control group and had the highest myelin formation. In a previous study, it was reported that the favorable environment for nerve regeneration was due to neural protective factors secreted from transplanted cells [46]. Given the high expression of genes related to cytoprotection (HSP-27, HSP-70) and anti-inflammation (IL-10, HO-1) in HS-treated MSCs, these neural protective factors in transplanted stem cells were considered to have changed the detrimental properties of reactive astrocytes, maximizing the growth-promoting properties.

Demyelination is a pathological process that occurs after SCI, and gray matter is lost faster than white matter [47]. Our data also revealed more loss of gray matter than white matter in all groups. However, compared with the other groups, the extent of myelin formation was high in both white and gray matter in HS-treated MSCs. Given that HSP-27 and HSP-70 are distinctly expressed in multiple sclerosis myelin, it has been confirmed that they play a role in protecting and repairing myelin [48]. Therefore, these results may indicate the protective and reparative effects of HSP on myelination. It should also be noted that the expression of beneficial genes increased in the HS-treated MSCs group, reducing the inhibitory capacity of reactive astrocytes and creating a permissive environment, which is in line with prior research [49]. The beneficial gene expression of HS-treated MSCs was demonstrated in the current study, where the expression of BDNF was high in HS-treated MSCs. There are reports that the expression of beneficial molecules such as BDNF creates a permissive environment for promotion of axonal regrowth [50,51]. As mentioned in a previous study [49], this permissive environment was considered to have highly expressed immature neuronal markers (β-III-tubulin) and neural stem/progenitor cell markers (nestin) in HS-treated MSC groups.

The HS-treated MSCs appear to have kept the reactive astrocytes at an appropriate level in the lesion, maximizing the effects of nerve protection and regeneration. This result was also corroborated by the degree of post-operative hindlimb recovery. The HS-treated MSC group showed faster and more improved functional recovery. Although we did not conduct an assessment of the neural circuits, they are thought to have promoted functional recovery by the surviving host neurons and transplanted cells, forming new synaptic connections in an indirect manner [2]. As previously reported [52,53], our results show that GFP-positive cells and β-III-tubulin positive cells remained in the area surrounding the injured spinal cord region. These results suggest that neural regeneration takes place in the periphery, rather than in the lesion core.

## 5. Conclusions

To improve the efficacy of stem cell treatment for SCI, stem cells must endure in a hostile environment of lesions. In this respect, HS-treated MSCs not only have cytoprotective effects in SCI environments but also transform the surrounding environment into one favorable for nerve regeneration. Therefore, the application of HS-treated MSCs to acute SCI in dogs could have beneficial effects such as neural protection and neuroregeneration in the hostile environment of acute SCI.

## Figures and Tables

**Table 1:** Primers sequence used for real-time quantitative PCR.

Target Gene (bp) (Accession Number)	Primer Sequence (5′-3′)	
	Forward (Tm)	Reverse (Tm)
GAPDH (105) (XM_025471882.2)	CATTGCCCTCAATGACCACT (58.16)	TCCTTGGAGGCCATGTAGAC (58.80)
OCT-4 (144) (XM_025418033.2)	AGCAGAAGAGGATCACCCTA (57.14)	GCCGCAGCTTACACATATTC (57.33)
SOX-2 (152) (XM_025451585.2)	AACCCCAAGATGCACAACTC (58.38)	CGGGGCCGGTATTTATAATC (55.84)
HSP-70 (125) (XM_025418242.2)	ACATCAGCCAGAACAAGCGA (59.96)	GAAGTCGATGCCCTCGAACA (60.11)
HSP-27 (199) (XM_025425872.2)	TAACTGGCAAGCACGAAGAG (58.20)	TCGAAGGTGACGGGAATAGT (58.16)
HO-1 (220) (XM_025461329.2)	CCAGTGCCACGAAGTTCAA (58.30)	TCTTGTGCTCTGCTGCCAAC (61.17)
IL-10 (120) (XM_025429742.2)	CCACGACCCAGACATCAAGAA (60.00)	TCCACCGCCTTGCTCTTATTC (60.41)
BDNF (244) (XM_025459817.2)	GCTGGCGGTTCATAAGGATA (57.46)	GTTTCCCTTCTGGTCATGGA (57.12)
CXCR-4 (127) (XM_025430622.2)	GAGCGGTTACCATGGAAGAG (58.06)	CGGTTGAAGTGAGCATTTTCC (58.07)
CCL7 (138) (XM_025466418.2)	CTCCGAACTGTGCCCTTCAG (60.67)	CCTGCGCCTCTCACATCT (59.10)

**Table 2:** Canines of Basso, Beattie, and Bresnahan score.

Score	Description
0	No observable hind limb (HL) movement
1	Slight movement of one or two joints
2	Extensive movement of one joint, or extensive movement of one joint and slight movement of one other joint
3	Extensive movement of two joints
4	Slight movement of all three joints of the HL
5	Slight movement of two joints and extensive movement of the third
6	Extensive movement of two joints and slight movement of the third
7	Extensive movement of all three joints in the HL
8	Plantar placement of the paw with no weight support
9	Plantar placement of the paw with weight support only when stationary, or occasional, frequent or consistent weight-supported dorsal stepping and no plantar stepping
10	Occasional weight-supported plantar steps; no FL–HL coordination
11	Frequent to consistent weight-supported plantar steps and no FL–HL coordination
12	Frequent to consistent weight-supported plantar steps and occasional FL–HL coordination
13	Frequent to consistent weight-supported plantar steps and frequent FL–HL coordination
14	Consistent weight-supported plantar steps, consistent FL–HL coordination, and predominant paw position is externally rotated when it makes initial contact as well as just before it is lifted off; or frequent plantar stepping, consistent FL–HL coordination, and occasional dorsal stepping
15	Consistent plantar stepping and consistent FL–HL coordination and no toe clearance or occasional toe clearance; predominant paw position is parallel to the body or internally rotated at initial contact
16	Consistent plantar stepping and consistent FL–HL coordination and toe clearance occurs frequently; predominant paw position is parallel or internally rotated at initial contact and externally rotated at liftoff
17	Consistent plantar stepping and consistent FL–HL coordination and toe clearance occurs frequently; predominant paw position is parallel or internal at initial contact and at liftoff
18	Consistent plantar stepping and consistent FL–HL coordination and toe clearance occurs consistently; predominant paw position is parallel or internal at initial contact and at liftoff. Trunk instability is present
19	Consistent plantar stepping and consistent FL–HL coordination and toe clearance occurs consistently during forward limb advancement; predominant paw position is parallel or internal at initial contact and at liftoff. Trunk instability is not observed

FL = forelimb; HL = hindlimb.

**Table 3:** Revised and modified Tarlov scales.

Item	Revised Scale	Modified Tarlov
Flaccid hind limbs	1	1
Tone in hind limbs	2	
Purposeful hind limb motion	3	2
Stands with assistance	4	
Stands unassisted	5	3
Limited ambulation	6	
Full ambulation	7	4
Climbs a 20° incline ramp halfway	8	
Climbs 20° incline ramp	9	5

## Supplementary Materials

The following are available online at https://www.mdpi.com/2076-2615/10/11/2164/s1, Video S1: Placement of the catheter with the contrast agent.
